# The effectiveness of financial incentives for smoking cessation during pregnancy: is it from being paid or from the extra aid?

**DOI:** 10.1186/1471-2393-12-24

**Published:** 2012-04-02

**Authors:** Eleni Mantzari, Florian Vogt, Theresa M Marteau

**Affiliations:** 1 Department of Psychology (at Guy's), Health Psychology Section, King's College London, 5th floor Bermondsey Wing, Guy's Campus, London SE1 9RT, UK

## Abstract

**Background:**

Financial incentives appear to be effective in promoting smoking cessation in pregnancy. The mechanisms by which they might operate however, are poorly understood. The present study examines how financial incentives for smoking cessation during pregnancy may work, by exploring pregnant women's experiences of trying to stop smoking, within and outside of a financial incentives scheme.

**Methods:**

Thirty-six (n = 36) UK-based pregnant smokers (n = 36), offered standard NHS Stop-Smoking Services, of whom twenty (n = 20) were enrolled in a financial incentives scheme for smoking cessation (n = 20) and sixteen (n = 16) were not, were interviewed about (i) their motivation to stop smoking, and (ii) the factors they perceived as influencing their quitting efforts. Framework Analysis was used to analyse the data.

**Results:**

Women in the two groups reported similar reasons for wanting to stop smoking during pregnancy. However, they described dissimilar experiences of the Stop-Smoking Services, which they perceived to have differentially influenced their quit attempts. Women who were incentivised reported using the services more than women who were not incentivised. In addition, they described the motivating experience of being monitored and receiving feedback on their progress. Non-incentivised women reported problems receiving the appropriate Nicotine Replacement Therapy, which they described as having a detrimental effect on their quitting efforts.

**Conclusion:**

Women participating in a financial incentives scheme to stop smoking reported greater engagement with the Stop-Smoking Services, from which they described receiving more help in quitting than women who were not part of the scheme. These results highlight the complexity of financial incentives schemes and the intricacies surrounding the ways in which they operate to affect smoking cessation. These might involve influencing individuals' motivation and self-regulation, changing engagement with and provision of support services, or a combination of these.

## Background

Smoking during pregnancy is a major cause of infant morbidity and mortality [1] and contributes greatly to health inequalities [2]. It causes up to 4,000 deaths per year in the UK from miscarriages and stillbirths, and leads to increases in preterm births, low birth-weight babies [3,4], sudden infant death, asthma and attention deficit hyperactivity disorder [4,5]. Despite these adverse consequences, many women fail to quit while pregnant, with at least 17% of mothers in the UK smoking throughout their pregnancies in 2005 [6]. Reducing the incidence of smoking during pregnancy has therefore become an important focus of health policies in the UK and elsewhere.

Existing interventions have been relatively successful in promoting smoking cessation during pregnancy [7,8]. A recently updated systematic review [9] found the most effective of these to involve the use of financial incentives for stopping smoking (financial incentives vs. other interventions: OR 0.73, 95% CI 0.66 to 0.82). Findings were based on results from four trials conducted in the USA [10-13] and were confirmed by a further meta-analysis of three of these [14]. The mechanisms by which financial incentives operate to influence behaviour, including smoking cessation during pregnancy, are, however, poorly understood.

The effectiveness of financial incentives in achieving behaviour change, including smoking cessation during pregnancy, might result from direct influences to individuals' motivation and self-regulation. These influences potentially enable people to overcome the costs and barriers associated with initiating the target behaviour and move them past the "threshold" needed to act. Specifically, incentives might operate according to learning theory principles, by linking the target behaviour, in this case smoking cessation, to a positively evaluated stimulus, such as money, thus strengthening the value associated with the target behaviour [15]. Additionally they might work by influencing individuals' outcome expectations, i.e. their valuation of the likely consequences of a behaviour [16], or by facilitating allocation of limited cognitive capacity in such a way as to achieve the now more highly valued altered behaviour [15].

The effectiveness of financial incentive schemes in changing behaviour might also result from indirect influences, mediated by changes to some aspects of the process involved in their delivery. For example, the provision of incentives requires contact between health professionals, who measure achievement of the target behaviour, and patients [17]. Incentives might therefore operate by increasing health professionals' engagement with patients or through the additional involvement required on behalf of the latter, such as attending clinics or undergoing particular tests, as part of assessing eligibility for a reward. In addition, they might influence behaviour through the contract-agreement, which specifies the conditions of exchange between behaviour and money, encompassed in their use [17], given that behavioural contracts have been shown to improve patients' adherence to health care activities, even in the absence of the exchange of money [18]. It is also possible however, that the effectiveness of financial incentives in achieving behaviour change might also result from an interaction between direct influences to individuals' motivation and self-regulation and indirect influences mediated by changes so certain aspects involved in the process of incentive delivery.

Understanding the mechanisms by which financial incentives influence behaviour is key to determining how to maximize their effectiveness [19] and for designing optimal incentive schemes. Research is therefore needed to illuminate the processes involved in producing their beneficial effect for smoking cessation during pregnancy. Given the lack of knowledge regarding the factors that are operating when financial incentives schemes are used, qualitative research has an important contribution to make. The present qualitative study attempts to explore these factors by examining and comparing the stop-smoking experiences of pregnant women who were incentivised for smoking cessation and of pregnant smokers who were not incentivised for cessation.

## Methods

### Design

This is a comparative qualitative study, based on semi-structured interviews aiming to identify differences between the experiences of pregnant smokers who were incentivised for cessation and of those who were not.

### Participants

**Figure 1:**
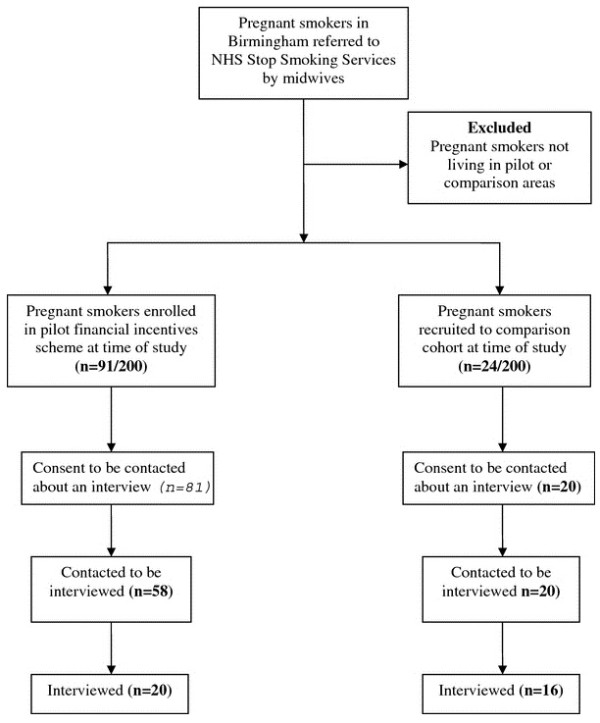
**Recruitment of participants**.

Participants were thirty-six (n = 36) pregnant smokers, twenty (n = 20) of whom were receiving financial incentives for smoking cessation (incentivised group). The remaining sixteen (n = 16) were only offered NHS Stop-Smoking treatment^1 ^(control group). Participants were recruited through an opportunistic sampling frame involving a population of 115 pregnant smokers living in the greater Birmingham area (Figure 1) who were referred by their midwives to the NHS Stop-Smoking Services during the period September 2009 to May 2010 and:

i. were enrolled in a pilot scheme of incentivising smoking cessation run by the Birmingham East & North Primary Care Trust (BEN PCT), (in partnership with the Young Foundation as part of the Healthy Incentives (HI) Partnership (http://www.healthyincentives.org.uk), or

ii. were eligible to be part of a comparison cohort, because they lived in areas selected as "comparison" areas.

Women enrolled in the financial incentives scheme were offered vouchers for quitting smoking. The offer of vouchers was dependent upon women's area of residence, i.e. whether they lived within the two pilot areas or not. Pilot areas were selected from the districts of Birmingham with the highest prevalence of smoking during pregnancy. The pilot financial incentive scheme aimed to enroll 200 pregnant smokers by the end of 2010 and to compare their smoking cessation rates against those of a comparison cohort of 200 women, recruited for evaluation purposes from parts of the PCT where financial incentives were not offered. Comparison areas were chosen by matching the pilot areas with two geographically similar districts with equivalent rates of smoking during pregnancy and comparable socio-economic composition. At the time the current study was conducted, 91 women were enrolled in the pilot financial incentives scheme, of whom 81 consented to be contacted for an interview. We aimed to recruit 20 of these women for the interview and achieved this with telephone calls to the first 58. Furthermore, 24 pregnant smokers had been recruited into the comparison cohort, of whom 20 consented to be contacted for an interview. All these women were contacted and 16 agreed to be interviewed.

Following the recommendations by Guest et al (2006) [20], as well as those by Kuzel (1992) [21] and Morse (1995) [22], this sample size was considered sufficient for achieving data saturation. Indeed, saturation of data for the themes of interest was achieved in both groups by the 15^th ^interview, suggesting that the group sizes were sufficiently large to capture the range of women's smoking cessation experiences.

The mean age of participants in the incentivised group was 28 (range: 19-43). The mean age of participants in the control group was also 28 (range 17-39). The majority of participants were of White-British origin, with one woman in the control group being of Indian decent and another in the incentivised group originating from Hong-Kong. Although, minority ethnic groups constitute approximately one third of Birmingham city's population (with the Pakistani being the largest minority group followed by the Indian) [23], women from minority ethnic groups are less likely to smoke compared to the general population [24]. Compared to white women, they are also less likely to smoke during pregnancy [25] and are less likely to set quit dates with the stop smoking services [26]. The majority of women in both groups were of a lower socio-economic class, as indicated by their Index of Multiple Deprivation Scores (incentivised group: 42.35; control group: 42.51) which are above the average for Birmingham^2^. Most women in both groups were unemployed. Of those who were employed, most held skilled non-manual and semi-skilled manual jobs in fields such as social care, maintenance and cleaning, automobile mechanics and law reinforcement. At the time of the interview, six (n = 6) women in the incentivised group and five (n = 5) in the control group had already delivered their babies. Furthermore, one individual in the incentivised group had miscarried. With regards to their smoking status, eight (n = 8) women in the incentivised group and four (n = 4) in the control group were smoke-free at the time of the interview. The remaining 24 individuals were still smoking.

### Procedure

Women in both groups were enrolled into the Stop-Smoking Services by the "Call to quit" call-centre (Birmingham's telephone line for information on local smoking cessation services). Women taking part in the financial incentives scheme for smoking cessation were asked by the call-centre's representative about their willingness to be contacted about the possibility of being interviewed about their experiences of quitting smoking. Women not taking part in the scheme were informed by a research midwife working for BEN PCT of the possibility of being interviewed. Women in both groups willing to be contacted about the study were approached by the interviewer (EM) via telephone. She informed them about the purpose of the research and enquired about their willingness to participate. At this point, all women were advised that they would receive £20 in cash to compensate for their time spent completing the study. A time and place [for a face-to-face interview] was arranged with those agreeing to be interviewed. The majority of participants chose to be interviewed in their homes, with one woman from the control group opting to be interviewed at her place of work. Ethical Approval for this study was granted by the NHS Birmingham, East, North and Solihull Research Ethics Committee, ref no 09/H1206/105.

### Interviews

Interviews were semi-structured and followed an interview schedule to elicit information on women's experiences of smoking cessation. The schedule was piloted with five pregnant smokers attending prenatal appointments at a London hospital.

Interviews lasted an average of 23 minutes and were digitally recorded. Upon their completion, women were thanked and received £20 in cash to compensate for the time spent participating in the interview.

### Data analysis

Anonymised interviews were transcribed verbatim and analysed using Framework Analysis [27] with the purpose of identifying and comparing the themes emerging in the accounts given by the two groups of women, with regards to i) their motivation for wanting to quit smoking, and ii) the factors they perceived as facilitating and inhibiting their quit attempts.

Framework Analysis was chosen because it provides a method of addressing specific research questions rather than for purely exploratory purposes. It consists of a matrix-based analytic method, which facilitates rigorous and transparent data management, such that all stages of analysis can be systematically conducted.

The analysis was conducted separately for each group of women. The resulting themes of interest where then tabled and compared to identify similarities and differences.

## Results

The themes emerging in the accounts given by the two groups of women, with regards to i) their motivation for wanting to quit smoking, and ii) the factors they perceived as facilitating and inhibiting their quit attempts, are presented below.

### Reasons for wanting to quit smoking during pregnancy

Women who were incentivised for smoking cessation and those who were not reported similar reasons for wanting to stop smoking during pregnancy, which were grouped under five themes: (i) Concern for baby, (ii) Feeling pressured, (iii) Financial issues, (iv) Concern for self and (v) Concern for existing children (Table 1).

**Table 1:** Reasons for wanting to quit smoking during pregnancy

Factor	Description	Incentivised Group	Non-Incentivised Group
**Concern for Baby**	Being pregnant and concerned about the possible consequences of smoking on the baby	***✓***	***✓***

**Feeling pressured**	**Internal Pressure**. Experiencing guilt for smoking while pregnant and feeling pressure from self not to do so	***✓***	***✓***
	
	**External Pressure**. Experiencing pressure from others not to smoke	***✓***	***✓***

**Financial issues**	**Expense of smoking**. Not affording to smoke and wanting to save money	***✓***	***✓***
	
	**Financial Incentives**. Wanting to get the vouchers	***✓***	***N/A***

**Concern for self**	Concern about the illnesses and physical damage (including damage to appearance) caused by smoking, about consequences on existing health problems (e.g. asthma) and wanting to increase energy levels	***✓***	***✓***

**Concern for existing children**	Being concerned about the consequences of smoking on the health of existing children, wanting to reduce the possibility of them becoming smokers because of exposure to smoking, and wanting to avoid causing children distress due to personal smoking-related health problems	***✓***	***✓***

The provision of *Financial incentives *emerged as a sub-theme of *Financial issues *in incentivised women's accounts of their motives for trying to quit:

"And then the vouchers give me incentive to, like, stop smoking" (Participant14, incentivised group)

This however was not discussed as a primary reason and was often described as an "added bonus" for already wanting to quit:

"... the vouchers and the incentives and I thought well, that's even better. That, to me, was an added bonus that wasn't a reason quit, that was just like a reward for actually going to them." (Participant26, incentivised group)

### Factors perceived as influencing the quit attempt

#### Perceived facilitators

The factors that were perceived as facilitating cessation efforts by women in both groups were grouped under two themes: (i) Endogenous factors and (ii) Exogenous factors. Facilitators described as deriving from within the self were classified as Endogenous, while those described as deriving from the environment were classified as Exogenous. Similar Endogenous factors were described by women who had been incentivised for cessation and those who had not. These were grouped under three sub-themes: (i) Awareness of the consequences of smoking and quitting; (ii) Dispositional factors (positive mood, motivational strength and personality characteristics); and (iii) Low addiction (Table 2).

**Table 2:** Factors perceived to facilitate smoking cessation attempt

Factor	Description	Incentivised Group	Non-Incentivised Group
***Endogenous***			

**Awareness of the consequences of smoking & quitting**	**On the baby's health**. Having knowledge or experience of the consequences of smoking on the unborn baby and thinking of potential harms	*✓*	*✓*
	
	**On resources**. Thinking that smoking leads to a waste of money and quitting efforts and experiencing the benefits of quitting on money and time	*✓*	*✓*
	
	**On personal health**. Thinking of the consequences of smoking on health and experiencing the physical benefits of quitting	*✓*	*✓*

**Dispositional factors**	**Personality**. Possessing traits associated with an increased ability to maintain focus and persist with efforts	*✓*	*✓*
	
	**Motivational strength**. Wanting to quit and being focused on quitting	*✓*	*✓*
	
	**Mood**. Being in a positive mood	*✓*	*✓*

**Low addiction**	**Lack of Cravings**. Not experiencing cravings for cigarettes and smoking	*✓*	*✓*

***Exogenous***			

**Availability of support**	*i) *Having friends, family and colleagues provide encouragement, praise, concurrent quitting, and prohibition of smoking or exposure to smoke	*✓*	*✓*

**Lack of exposure to smoke**	Lack of smoking in immediate environment and deliberately avoiding smoking situations	*✓*	*✓*

**Lack opportunity to smoke**	Decreased opportunities to smoke due to prohibition of smoking in certain places and around certain people, embarrassment of smoking in public, existence of health issues or preoccupation with other matters	*✓*	*✓*

**Stop Smoking Services**	**Receiving support& advice**. Being provided with support by speaking to smoking cessation counsellors and receiving information and advice	*✓*	*✓*
	
	**NRT**. Receiving NRT	*✓*	*✓*
	
	**Receiving feedback**. Getting feedback on progress, either verbally from members of the services, or by viewing improved CO levels	*✓*	***×***
	
	**Being monitored**. Having CO levels checked by the Stop-Smoking Services	*✓*	***×***

**Financial incentives**	Getting the vouchers	*✓*	*N/A*

Women in both groups also described comparable Exogenous factors as facilitating their efforts, which were grouped under five sub-themes: (i) Availability of support; (ii) Lack of exposure to smoke; (ii) Lack of opportunity to smoke; (iv) Stop Smoking Services; and (v) Financial incentives (Table 2). Their accounts differed, however, with regards to the dimensions that emerged in relation to one of the Exogenous factors, namely the Stop-Smoking Services. Although participants in both groups described the perceived beneficial effects of *Receiving support and advice *from the services and of the *Nicotine Replacement Therapy *that was provided by the services, incentivised women discussed the former more consistently and at a greater length than did non-incentivised women. Incentivised women additionally described the motivating experience of *Being monitored*:

"I think having that knowing that he was going to check what, what we were... the intake and stuff that was kind of the, the bit that was making me not want to smoke as well because it was like for the test..." (Participant02, incentivised group)

Specifically, women in this group described how having their carbon monoxide levels checked made them not want to smoke, out of the need to prove their abstinence:

"if I go to the chemist I have to prove to the pharmacist that I have cut down... it's a bigger goal" (Participant36, incentivised group)

This need appeared related to their fear of being judged for smoking during pregnancy:

"I knew that I'd got to go and check in, it's what, it's what that person would think of me I'm pregnant and I'm smoking and they'll going to know that I'm smoking. So it was that, having that support because I knew I'd have to face somebody. And I guess it was that being judged by..."(Participant26, incentivised group)

It also appeared to have arisen from their fear of being told off for not trying to quit:

"So I was constantly thinking about keeping my carbon monoxide levels down so I don't get into trouble... I thought it was like I keep smoking like my five/six a day then my carbon monoxide levels will either stay the same or go up a little bit. And it would be like, "You're not trying to quit why should I bother with you because you're not even participating". Do you know what I mean?" (Participant20, incentivised group)

Furthermore, it appeared to be associated with women's desire to avoid disappointing the smoking cessation counsellors:

"... they was very good. And I think it was going to somewhere like that every week that you didn't want to go and say, "I smoked." ((laughs)) You know it helped you... You didn't want to feel like I'd let it down or yeah (Participant25, incentivised group)

*Being monitored *was closely related to the sub-theme *Receiving Feedback*, which was also perceived by incentivised women as having a beneficial effect on their smoking cessation efforts:

"For me to be tested and everything is good because and it kind of makes you feel good when it comes up like that and they're like "Oh well done."" (Participant30, incentivised group)

In fact, *Receiving Feedback *was described as a consequence of *Being Monitored*: witnessing improved carbon monoxide levels and/or receiving related praise from the smoking cessation counsellors was perceived to increase confidence and was thus perceived as facilitating efforts:

"It's just more of a moral support I think really and checking your carbon levels and once you realise you've done good, you know, it boosts your confidence to keep, keep not smoking, do you know what I mean?" (Participant32, incentivised group)

These differences in experiences may be related to the observation that women in the control group were less engaged with the services, regardless of the fact that access was equal across the two groups: Whereas all women in the incentivised group had used the Stop-Smoking Services at least once, some individuals in the control group had failed to attend even their first appointments:

"So have you used the services this time round?"(Interviewer)

*"Not as yet - no" (Participant21, control group)*.

Had non-incentivised women used the services, their experiences might have been more similar to those of incentivised women, given that service delivery was meant to be identical across the two groups, with the exception of voucher provision. Indeed, when asked how being monitored each week would potentially influence her attempt to stop smoking, one woman in the control group who had not attended the services reported:

*"No I think that sounds good... Because it's, it's actually assessing you isn't it? You're not going to want to turn up there say you've not stopped smoking.... I think that would help me.... Because it's putting a little bit of pressure on me, it's pushing me a little bit... Because you want to do it anyhow and I suppose like somebody watching you constantly that's what it's like isn't it? *(*Participant35, control group*)

This differential engagement with the services seems related to the offer of *Financial Incentives *which appears to have motivated incentivised women to attend the services:

*"I wouldn't have bothered going all the way to the doctors because at the beginning of your pregnancy and that you don't want to go out the house anyway because you're feeling sick and you're heavy and frumpy, and it just seems like a long way to go for nothing just to blow into a thing. With the vouchers it's like you're getting paid... rewarded to go there" (Participant14; incentivised group)*.

Indeed, the *Financial Incentives *were perceived as facilitating cessation attempts:

"the vouchers give me incentive to like stop smoking... So the vouchers have helped yeah because I'm thinking it's not that worth risking." (Participant14, incentivised group)

The vouchers appeared to have achieved this by providing a goal to work towards and a focus for resisting urges to smoke:

"I feel like I need another one [cigarette] I sort of sit there and think to myself well if I have this one it's going to mess me up getting my vouchers for my kids.... I won't because I'll just think well I've got the vouchers to look forward to" (Participant16, incentivised group)

An alternative explanation for the absence of the aforementioned sub-themes from the accounts of non-incentivised women is that whereas monitoring in the incentivised group was conducted routinely due to attainment of the vouchers being contingent upon the results of such monitoring, monitoring in the control group was inconsistent. This accords with the accounts of two women in the control group, one of whom was not monitored and another who exceptionally, was:

*"They don't really monitor you... They only do it, they only did it the once" *(*Participant28, control group*).

*"I think that was the most useful thing and knowing that you were going back the following week and that it had to be good because there was a quantifiable way of seeing if you'd been sticking to the routine." (Participant13, control group; 28:20-23)*.

#### Perceived inhibitors

Similarly to the perceived facilitators, the factors that were perceived to inhibit cessation efforts, both by women who were incentivised and those who were not, were grouped under two themes: (i) Endogenous and (ii) Exogenous factors. Obstacles described as deriving from within the self were classified as Endogenous, while those described as deriving from the environment were classified as Exogenous. Similar Endogenous obstacles were described by women who had been incentivised for cessation and those who had not. These were grouped under four sub-themes: (i) Disregarding the consequences of smoking and quitting; (ii) Dispositional factors, (negative mood, lack of motivation strength and personality characteristics); (iii) Perceived benefits of smoking; and (iv) Addiction (Table 3).

**Table 3:** Factors perceived to inhibit smoking cessation attempt

Factor	Description	Incentivised Group	Non-Incentivised Group
***Endogenous***			

**Disregarding the consequences of smoking & quitting**	**On the baby's health**. Discounting the harm of smoking because of having experienced disconfirming situations. Also discounting harm because of reduced cigarette consumption or because of inability to visualise baby and disregarding the benefits of quitting at advanced pregnancy stage	*✓*	*✓*
	
	**On personal health**. Blocking out personal health concerns and disregarding harms of smoking due to lack of relevant experience or by dissociating self from smokers with health problems	*✓*	*✓*

**Dispositional** **factors**	**Personality**. Possessing traits associated with a decreased ability to maintain focus and an increased likelihood of giving in to temptations	*✓*	*✓*
	
	**Lack of motivation**. Not really wanting to quit because of enjoying smoking or not considering quitting important	*✓*	*✓*
	
	**Mood**. Being in a negative mood	*✓*	*✓*

**Perceived benefits** **of smoking**	**To deal with stress**. Thinking that smoking helps with stress and using it to calm nerves down	*✓*	*✓*
	
	**To deal with boredom**. Smoking when bored	*✓*	*✓*
	
	**To control weight**. Thinking that smoking helps control weight and that quitting would result in weight-gain	*✓*	*✓*
	
	**For social inclusion**. Feeling left out when not smoking and using smoking for social inclusion	*✓*	*✓*

**Addiction**	**Habit & Associations**. Associating smoking with certain times of the day and being used to smoking in certain contexts	*✓*	*✓*
	
	**Cravings**. Experiencing cravings for cigarettes and smoking	*✓*	*✓*

***Exogenous***			

**Lack of social support**	Not receiving encouragement or praise, being told not to smoke and not having non-smoker peers to set example	*✓*	*✓*

**Exposure to smoke**	Being exposed to smoke in the immediate environment	*✓*	*✓*

**Availability of cigarettes & opportunity to smoke**	Smoking in situations that allow doing so, such as in the absence of certain people or when cigarettes are accessible	*✓*	*✓*

**Stop Smoking Services**	**Lack of Support& Advice**. Being judged, not being listen to, not being given sufficient explanations and advise, not being followed-up and lacking attention and individualised support	*✓*	*✓*
	
	**NRT provision problems**. Not receiving the appropriate NRT	***×***	*✓*
	
	**Lack of expertise**. Lack of experience regarding smoking cessation in general and during pregnancy	***×***	*✓*
	
	**Accessibility issues**. Service not being local, waiting long to get an appointment or getting appointments at inconvenient times	*✓*	*✓*

**Financial incentives**	Problems with getting the vouchers	*✓*	*N/A*

Furthermore, women in both groups reported similar Exogenous factors as compromising their efforts, which were grouped under five sub-themes: (i) Lack of support; (ii) Exposure to smoke; (ii) Availability of cigarettes and opportunity to smoke; (iv) Stop Smoking Services; and (v) Financial incentives (Table 3). Their accounts, however, differed with regards to the sub-themes that emerged in relation to one of the Exogenous obstacles, namely the Stop Smoking Services.

Specifically, although participants in both groups described the perceived detrimental effects of the *Lack of Support and Advice *from the services and of the *Accessibility Issues*, non-incentivised women described the adverse effects of not receiving the appropriate Nicotine Replacement Therapy (NRT). This was perceived by women in this group as differentially affecting their cessation efforts and was mentioned as resulting from a lack of information on behalf of the services regarding the treatments allowed during pregnancy:

"... she gave me the patch where I wanted the highest patch that I could have because I've been smoking 20 24/7, they actually told me the most I could have was a 20 mg patch, which now I've been told by the midwife that's not true.... The patch didn't seem to be working. And then when I told my midwife it didn't work and she said it was, erm, that I could have more than a 20 mg patch. Where I'd got told that was all I could have... I was pregnant I wasn't allowed the highest dose I could have was the 20 mg patch... I wouldn't be smoking now If the pharmacist had give me the right amount" (Participant09, control group)

This NRT-provision problem was also discussed in relation to the services' lack of suggestions regarding alternative aids for women who were experiencing side effects with their existing treatment:

".... patches... because I've got eczema...... and they irritate my skin... No I went back, erm, and I tried the inhalers, but I didn't like them, they give me a sore throat and I didn't like when you suck on them you get a nasty taste in your mouth... And I have tried the gum but I don't like them they sort of burn your tongue and that... So I like, sort of run out of options. I didn't know what else I could try really..." (Participant07, control group; 8:16-24; 9:1-9)

It also seems to have stemmed from the specific prescription protocols adopted by the services:

I remember running out [of lozenge] not being able to get an appointment so... Basically my doctor... you'd phone at half eight in the morning it's engaged for ages. By the time you get through you can't get an appointment but now they've changed the rules. The doctor I went to see him last time I said, "Look please I can do it on... it's going to take me a month to get an appointment with your smoking nurse here" and I said, "can't you just give me the prescription now while I'm waiting?" But he wouldn't."(Participant34, control group; 18:1-18)

Not receiving the appropriate NRT appears associated to smoking cessation counsellors' lack of expertise, which was described as an additional factor inhibiting the efforts of non-incentivised women:

"I said to her, erm, er, yeah about me being pregnant and still carrying the lozenges she's like "Yeah." I said I've got patches at home can I still use them, like can I start on them again rather than give me more, they're from last year they're still in date though? And she said, "I've never dealt with a pregnant woman before."" (Participant34, control group)

This lack of expertise was perceived as generalised and not only in relation to smoking cessation during pregnancy:

"Actually she was actually reading off the form, so it wasn't like she knew it, she was reading it from a book when I kept signing it saying... And she was reading from there about the cravings and how the patch works and if I need to go in and talk to them. She wasn't saying it off her head, she was reading it off a form...[]... I think that's... she didn't know but really that's wrong because they're a pharmacy. Because they're a Stop Smo--... how you can stop smoking they should have all the right information. So I think someone needs to go to them and see if they have got the right information." (Participant09, control group)

The above issues were only raised by women who were not participating in the incentive scheme for cessation. Given that access to NRT was meant to be identical across the two groups, this finding raises questions regarding whether it reflects differences in perception, or actual differences in service provision. These possibilities will be discussed in the next section.

Incentivised women were unique in their descriptions of the inhibiting effects of encountering problems with obtaining the vouchers, which they perceived as having compromised their smoking cessation attempt:

"Well it didn't work very well because the first week we went my voucher came, but it didn't come to my address it came to another address and they sent it on. And then the next time I went to the chemist for the next test I didn't tell him that he hasn't got my address right, and my voucher never came.... that put me off then" (Participant19, incentivised group)

## Discussion

Women in the two groups reported comparable reasons for wanting to stop smoking during pregnancy. While citing broadly similar factors as influencing their quit attempts, their accounts differed with regards to their experiences of the Stop-Smoking Services. Women who were incentivised described the motivating experience of being monitored and receiving feedback on their progress. Non-incentivised women reported problems receiving the appropriate Nicotine Replacement Therapy, which they described as having a detrimental effect on their cessation efforts.

### Reasons for wanting to stop smoking

Although women in the two groups reported similar motivations for trying to stop smoking, the accounts of incentivised women differed with regards to the mention of financial incentives. Attainment of the incentives by those in the incentivised group, however, was not described as a primary reason for attempting to quit smoking, but was referred to as an "added bonus" for doing something they were already motivated to do. The incentives therefore were not described as having an influential role in women's decisions to stop smoking. This is consistent with the findings of a recent investigation showing that the majority of quitters, among non-pregnant smokers, did not consider incentive-attainment as a main reason for quitting smoking [28]. There are three possible explanations for this finding.

Firstly, it may reflect an actual failure of incentives to influence women's motivation to stop smoking. The value of incentives offered in the current scheme was considerably smaller (more than ten-fold less) than that offered in the trials from which there is evidence of effectiveness [11-13]. They were also offered as fixed sums at fixed periods of time. Consequently, they may have been too small or offered in a way unlikely to influence motivation or shape new behaviours. Initial impressions of the scheme's effectiveness, however, do not appear to support this explanation: a larger number of women from the incentivised group compared to the non-incentivised group were referred to the Stop-Smoking Services. Although this could in part be attributable to midwives' differential engagement with women from each group, it may also reflect incentivised women's greater willingness to be referred to the services and thus greater motivation to stop smoking.

A second possible explanation for the aforementioned finding is that women were not aware of the effect financial incentives had on their motivation to stop smoking. Indeed, people are often unaware of the processes underlying their thoughts and motivation for their behaviours [29-31]. It is therefore possible that financial incentives influenced women's motivation outside their conscious awareness. The mechanisms by which this could occur are unclear. One hypothesis is that incentives work through increasing positive affect, which can be induced by the provision of money [32] and is considered to have a fundamental role in non-conscious motivation [33].

The third explanation for the aforementioned finding is that women were aware of the effect financial incentives had on their motivation to stop smoking but were unwilling to admit it. Smoking during pregnancy is surrounded by social stigma. The majority of people are critical of pregnant smokers and view smoking during pregnancy as an indication of women not taking the responsibilities of motherhood seriously [34]. As such, pregnant women often perceive pressure to stop smoking [35], with people feeling that they should do so for medical and social reasons [36]. The use of financial incentives for health promotion is also surrounded by negative attitudes, with people often finding such interventions unacceptable [37] and arguing that individuals should not be paid to do things they should do anyway [38]. Taken together these negative attitudes may have lead women in the present study to feel pressure to focus more on the health reasons for quitting smoking, such as for the health of their baby and underplay the influence of incentives.

### Factors influencing quit attempts

While women in the two groups perceived broadly similar factors as having influenced their quitting efforts, their accounts differed with regards to their dissimilar experiences of the Stop Smoking Services. Incentivised women described the motivating experience of being monitored and receiving feedback on their progress. Non-incentivised women on the other hand described the detrimental effect of not receiving the appropriate Nicotine Replacement Therapy (NRT). There are at least two possible explanations for these differences.

Firstly, given that access to the services and their delivery was meant to be identical across groups, findings may represent a difference in perception that is not reflected in actual delivery of the services. Specifically, differences in women's levels of engagement with the services may have influenced how they perceived them. Repeated exposure to novel stimuli increases liking [39]. Accordingly, incentivised women's greater use of the services, which appeared related to the provision of incentives, may have led them to focus more on the services' positive aspects. Similarly, the lack of engagement by non-incentivised women may have led them to focus on the negative aspects. Exposure can also have positive effects on affect [40,41] which has been shown to influence thinking, and the evaluation of events [42-44] as well as attitude formation [45]. The provision of money has also been shown to induce positive affect [32]. Consequently, differences in perception might have resulted from differences in positive affect. Furthermore, given that affect generated by one stimulus can be transferred to another [46,47], the positive affect resulting from incentive-attainment may have generalised to the context in which this occurred, i.e. the Stop-Smoking Services, thus leading incentivised women to perceive the services more positively. If differences in support are perceived, rather than actual, and reflect a differential engagement with the services, then the use of incentives might be effective to the extent that they increase pregnant smokers' involvement with the services.

A second explanation for the aforementioned perceived differences is that they may reflect an actual difference in women's experience of the services. This may have resulted from differential engagement with the services, related to the provision of incentives, as well as differential delivery of the services. The latter may have resulted regardless of the intention to keep the services identical across groups. The incentive scheme was not randomised across services, but rather was provided in different parts of a geographical area in England. It is therefore possible that service delivery differed in these areas. Indeed, it is accepted that Services vary in the types of interventions they choose to provide and their approaches to delivery depending on local circumstances and patients' preferences [48,49]. Although guidelines exist with regards to the elements all interventions should include, such as CO monitoring and delivery of progress related feedback, [49] provision of these varies greatly within the NHS Stop Smoking Services [50]. Differences may have also been related to the provision of financial incentives. Incentivised women appeared to be using the services more as a result of the incentives. This greater engagement may have given women in this group more of an opportunity to experience service-related support. Furthermore, because voucher delivery was contingent upon biochemically confirmed smoking cessation, monitoring of smoking behaviour and provision of related feedback from the services might have been more regular for incentivised women. This would explain the absence of these themes from the accounts of non-incentivised women. Moreover, being involved in a programme specifically aimed at pregnant smokers may have led smoking cessation counsellors included in the financial-incentives scheme to receive more education and training about the NRT aids allowed during pregnancy. Absence of such training, due to the lack of involvement with a scheme designed for pregnant smokers, could explain non-incentivised women's experiences of problems with NRT-provision. Indeed, women in this group discussed these problems, in relation to service providers' inadequate knowledge and expertise.

If differences in the delivery of the Stop Smoking Services are actual rather than perceived and if the incentives scheme is shown to be effective in promoting smoking cessation, then one possible explanation would be that its impact is due wholly or in part to increased levels of support from the services, provided in the form of monitoring, progress-related feedback and/or delivery of appropriate NRT. Given the exploratory nature of the current study, in addition to the lack of a formal evaluation of the effectiveness of the incentive scheme, this hypothesis has not yet been tested. Further research is necessary to establish whether the potential effectiveness of financial incentives is indeed mediated by increased levels of support from the services. If this is the case, it may be possible to improve smoking cessation rates by furthering service providers' training and ensuring delivery of regular monitoring and progress-related feedback, rather than providing incentives. However, while there is some evidence to suggest the effectiveness of NRT in reducing smoking in pregnancy [14], biochemical risk assessment, including CO measurement and feedback, does not appear to aid smoking cessation [51]. This finding could be taken as an indication that incentivised women's perceptions of the beneficial influence of monitoring and feedback provision, in reality, may not have necessarily affected their cessation success. Further research is necessary to elucidate the role of service-support in the effectiveness of financial incentives for smoking cessation during pregnancy and to clarify the role of other potentially important variables in the mediation of the impact of financial incentives for smoking cessation during pregnancy.

### Strengths and limitations

The present study has certain important strengths. First, it is the first investigation attempting to determine how financial incentive schemes for smoking cessation during pregnancy may have their effects. Consequently, it is the first to explore the experiences and perceptions of pregnant smokers who have been incentivised for cessation and compare them with those of pregnant smokers not receiving incentives. This comparative design allowed for identification and exploration of the factors that are potentially important for smoking cessation during pregnancy. Finally, the strength of this study also lays in the size of its sample: it is one of the largest interview-based studies of pregnant smokers, focusing on the accounts of thirty-six women. This is important as pregnant smokers are an extremely difficult group to recruit and study.

The current study has certain limitations that restrict assessment of how such incentives may be having an effect. First, the qualitative, exploratory nature of the study does not allow for causal relationships to be established. Second, as mention previously, the incentives scheme is pending formal evaluation and its effectiveness has yet to be established. At the time the interviews were conducted few women in either group had stopped smoking, thereby precluding comparisons within and between groups between quitters and non-quitters.

## Conclusion

Regardless of the above limitations, the findings presented here highlight certain important issues about incorporating financial incentives for smoking cessation during pregnancy into the NHS Stop-Smoking Services. These include the need to be cautious about attributing the effects of financial incentives schemes to incentives *per se*, given that such schemes are complex behavioural interventions that might operate through one or more of various pathways, including by increasing individuals' motivation and self-regulation, by changing their engagement with and provision of support services, or a combination of these.

## End notes

^1^The NHS Stop Smoking Services were set up in England in 1999 to provide assistance to smokers motivated to quit. Services are provided in group or individual sessions, depending on local circumstances and patient preferences. Services vary in the types of interventions they provide and in their approaches to delivery [48]. Guidelines however, specify that Nicotine replacement therapy (NRT), Champix (varenicline) and Zyban (bupropion), in combination with intensive behavioural support should be offered to all smokers using the services. Other elements services should include are: monitoring of carbon monoxide (CO) levels and feedback of results [49]. The guidelines also specify that pregnant smokers should be offered the full range of services, including biochemical verification of smoking status and nicotine replacement therapy [47].

^2 ^According to the West Midland Regional Observatory the most deprived area within the West Midlands is Birmingham with 39.63% of its Lower Layer Super Output Areas (LSOAs) ranking in the worst 10% in England and an average IMD score of 38. 41.

## Competing interests

The authors declare that they have no competing interests.

## Authors' contributions

EM collected and analysed the data for this study and drafted the manuscript. FV contributed in analysing and interpreting the findings and participated in drafting the manuscript. TM designed the study's method and participated in interpreting the findings and drafting the manuscript. All authors read and approved the final manuscript.

## Pre-publication history

The pre-publication history for this paper can be accessed here:

http://www.biomedcentral.com/1471-2393/12/24/prepub
